# Discrepancy in diagnosis and characteristics of odontogenic cystic lesions in mixed dentition period; a retrospective study

**DOI:** 10.1016/j.jobcr.2025.09.028

**Published:** 2025-10-01

**Authors:** Umer Mukhtar, Rigzen Tamchos, Manoj kumar Jaiswal, Sadia Iqbal

**Affiliations:** Oral Health Sciences Center, Postgraduate Institute of Medical Education and Research, Chandigarh, India

**Keywords:** Discrepancy in diagnosis, Odontogenic cyst, Mixed dentition

## Abstract

**Background:**

There is an ambiguity in the correct diagnosis of odontogenic cystic lesions in mixed dentition period. So, present study was planned to assess diagnostic discrepancies and their potential impact on treatment strategies in pediatric odontogenic cysts.

**Material method:**

The data of the patients was retrieved from the digital records of patients from January 2014 to January 2024. After screening of the digital records, 61 cases were selected for screening, for demographic details, various clinical characteristics, radiographic investigations (OPG, CBCT etc.). For the calculation of the discrepancy between clinical and histopathological diagnosis of the radicular cyst, dentigerous cyst, and odontogenic kerato-cyst the Discrepancy Index was calculated.

**Results:**

The results revealed that 61 cases of various cystic conditions were identified. Among them, the dentigerous cyst constituted 14.7 % (9cases), radicular cyst constituted 42.6 % (26cases), and Odontogenic kerato-cyst constitutes 27.86 % (17 cases) with the mean age (in years) of reporting 9.55 ± 3.16, 9.00 ± 2.79, and10.06 ± 2.43 respectively. The odontogenic cysts were commonly found in mandibular posterior region. In patients with dentigerous cysts, 44.44 % had a history of extraction of primary teeth, 55.55 % had decayed/pulpectomised teeth. Among them the maximum discrepancy index was observed between dentigerous cysts and Odontogenic kerato-cysts i.e., 50 %, followed by radicular cyst and dentigerous cyst or vice-versa (21.42 %).

**Conclusion:**

Despite the difficult diagnosis of dentigerous cyst, radicular cyst and OKCs in mixed dentition, cystic lesions should be examined thoroughly and diagnosed carefully. Misinterpreting a cyst as a tumor, could lead to aggressive surgical intervention when a less invasive approach would suffice.

## 1 Introduction

Odontogenic cysts are pathological lesions frequently affecting the jaws of the maxillofacial region and may be either inflammatory or developmental in origin. Cysts associated with odontogenic epithelium arise from epithelial cells of the dental follicle or the remnants of odontogenic epithelium such as reduced enamel epithelium, rests of Malessez, Hertwig's epithelial root sheath, or rests of Serres.^1^ Among the cystic lesions, most commonly occurring is radicular cyst, associated with primary mandibular molars and frequently observed in early mixed dentition periods with an equal distribution among genders. The cause of radicular cysts is associated with necrotic pulp tissue, usually resulting from dental caries or dental trauma. Microorganisms from the necrotic pulp tissue in the root canal system and the bacterial toxins along with inflammatory mediators traverse into the periapical region and furcation area, thereby causing a cascade of interactions between epithelial and stromal cells. This low-grade inflammation ultimately triggers the stimulation and proliferation of epithelial cell rests, eventually forming a radicular cyst.^2^^,^^3^ Dentigerous cysts develop from dental follicles of unerupted teeth.^4^ Dentigerous cysts are found in the crown portion of impacted teeth, whereas radicular cysts are observed in the roots of infected teeth.^5^^,^^6^

Though these two cystic lesions are easy to distinguish based on clinical and radiographic findings when present in permanent dentition. However, in the mixed dentition stage, these two cystic lesions, when associated with carious primary teeth, are difficult to clinically diagnose, as the roots of primary teeth and the developing tooth buds of premolars are in proximity anatomically thereby complicating the clinical diagnosis.^7^^,^^8^ The odontogenic keratocyst (OKC)which show a close resemblance with the dentigerous cyst based on the presentation. The odontogenic kerato-cyst (OKC) is a developmental cyst that originates from remnants of the dental lamina within the jawbones.^17^ Several studies have reported a preference for males^9^, ^10^, ^11^ with an incidence steeping around the third decade.^12^ Keratocysts are characterized by a high recurrence rate, specific histological features, and aggressive clinical behavior. A study conducted by Naida Zaib et al., 2023 on the Discrepancy Index between Clinical and Histopathological Diagnosis of Oral neoplastic and non-neoplastic lesions.^13^ However, the literature is lacking in studies who have seen the discrepancy between the clinical and histopathological diagnosis of cystic lesions in the pediatric age groups. The correct clinical diagnosis of such cystic lesions is of the utmost importance and misdiagnosing dental cystic lesions can lead to several negative clinical consequences, including the possibility of incorrect treatment, prolonged symptoms, and potential damage to adjacent teeth and bone. It can also lead to the unnecessary or inadequate treatment of the affected area. Thus, this study aimed to assess the frequency of diagnostic discrepancies between clinical and histopathological diagnoses of odontogenic cysts in mixed dentition and their impact on treatment decisions. The null hypothesis for this study was that no discrepancy exists between the clinical and histopathological diagnosis of cystic lesions.

## 2 Material methodology

### 2.1 Study design

**Fig. 1:**
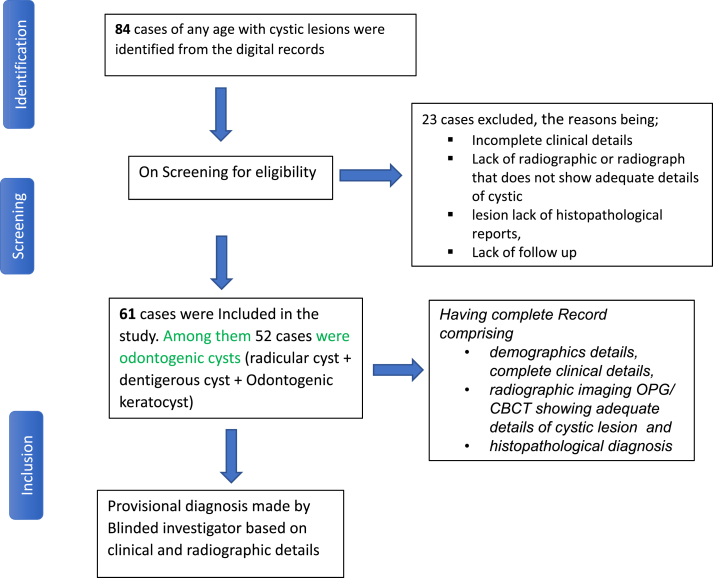
Flow diagram of retrospective case records through the study (STROBE statement).

The present retrospective study was conducted in the department of Pediatric and Preventive Dentistry, after taking ethical approval from the Institutional Ethical Committee. The study was configured using the STROBE guideline statement [Fig. 1].^14^ The data of the patients was retrieved from the digital records of patients visiting the department from January 2014 to January 2024. Children of age till 16 years with intraosseous cystic lesions with complete records comprising clinical findings, radiographic investigations, differential and provisional diagnosis made by unit by pedodontist with 10 years of experience (based on clinical radiographic and fluid aspiration), intervention and follow-up, were included in the study. For the provisional diagnosis of cystic lesions, the following criteria was followed;

**Table 1:** Distribution of demographic Characteristics of various lesions in pediatric patients and association of them with the gender.

Definitive diagnosis	Number of cases	Mean Age (Years)	p-value	Gender	
	N = 61			Male	Female
Dentigerous cyst	9 (14.7 %)	9.55 ± 3.16	p = 0.20 (ANOVA test)	7	2
Radicular cyst	26 (42.6 %)	9.00 ± 2.79		19	7
OKC	17 (27.86 %)	10.06 ± 2.43		1	2
AOT	3 (4.9 %)	12.33 ± 1.69		15	2
CGCG	1 (1.6 %)	12		0	1
COF	1 (1.6 %)	11		1	0
Fibrous dysplasia1	1(1.6 %)	11		0	1
MNTI	2 (3.27 %)	3 ± 1 months		1	1
Ameloblastoma	1(1.6 %)	15		0	1
Total	61(100 %)			44(72.13 %)	17(27.86 %)
				*Chi-Square =13.23*	
				*Degrees of freedom =8*	
				*p-value=0.1041*	

∗2.59 times more in males as compared to female.

**Table 2:** The Clinical characteristics observed in commonly seen odontogenic cysts in the pediatric patients in mixed dentition state which include chief complaint, location of swelling/pain, and status of primary tooth/teeth.

Clinical Features		Radicular Cyst	Dentigerous cyst	Odontogenic keratocyst N = 17
		N = 26	N = 9	
Chief Complaint	Pain	2 (7.6 %)	0	2(11.77 %)
	Swelling	14 (53.84 %)	6 (66.66 %)	10(58.82 %)
	Pain and swelling	10 (38.46 %)	3 (33.33 %)	5(29.41 %)
Location	Maxillary Posterior	4 (15.38 %)	3 (33.33 %)	4(23.52 %)
	Maxillary Anterior	3 (11.53 %)	0	1(5.88 %)
	Mandibular Posterior	17 (65.38 %)	6 (66.66 %)	7(41.17 %)
	Mandibular Anterior	1 (3.8 %)	0	5(29.41 %)
Status of associated primary teeth	History of Extracted	5 (19.23 %)	4 (44.44 %)	3(17.64 %)
	Decayed/Pulpectomized	21 (80.76 %)	5 (55.55 %)	6(35.29 %)
	Sound	0	0	8(47.05 %)

**Table 3:** The radiographic characteristics found in commonly seen odontogenic cysts in pediatric patients in mixed dentition state which comprises, type of investigation, locularity and borders of lesion, status of succednous tooth, and size of lesion.

Radiographic features		Radicular Cyst N = 26	Dentigerous cyst N = 9	Odontogenic keratocyst N = 17
Type of Investigations	OPG	19 (73.07 %)	4(44.44 %)	7(41.17 %)
	CBCT	5 (19.23 %)	4(44.44 %)	7(41.17 %)
	OPG + CBCT	2 (7.69 %)	1 (11.11 %)	3(17.64 %)
Radiographic Borders	diffused	16 (61.53 %)	4(44.44 %)	2(11.76 %)
	smooth	10 (38.46 %)	5(55.55 %)	7(41.17 %)
	corticated	0	0	8(47.05 %)
Locularity	Unilocular	25 (96.15 %)	8(88.88 %)	14(83.35 %)
	Bilocular	0	1(11.11 %)	1(5.88 %)
	Multilocular	1 (3.84 %)	0	2(11.76 %)
Status of succedaneous tooth/teeth	Absent	2 (7.69 %)	0	0
	involved but not displaced/resorbed	3 (11.53 %)	0	2(11.76 %)
	Displaced	21 (80.76 %)	8(88.88 %)	15(88.23 %)
Size of cystic lesion Maximum diameter of radiolucency	Class I = 10–20 mm	13 (50 %)	2(22.22 %)	3(11.64 %)
	Class II = 20–30 mm	11 (42.30 %)	2(22.22 %)	9(52.94 %)
	Class III >30 mm	2 (7.69 %)	5(55.55 %)	5(29.41 %)

**Table 4:** Discrepancy between provisional and definitive diagnosis of radicular cystic lesions, dentigerous cyst, and odontogenic kerato-cyst involving succedaneous teeth involved in mixed dentition period and type of surgical intervention rendered.

Clinical diagnosis	Histopathological diagnosis	No. of cases	Discrepancy Index (DI)
Radicular cyst	dentigerous cyst	3	21.42 %
dentigerous cyst	radicular cyst	3	21.42 %
dentigerous cyst	odontogenic kerato-cyst	7	50 %
odontogenic kerato-cyst	dentigerous cyst	1	7.14 %
Total	52	14(26.92 %)	

The discrepancy index was calculated using the following formula.

$Discrepancy\;Index\left(DI\right)=\frac{Number\;of\;incompatible\;cases}{Total\;number\;of\;cases\;in\;the\;study}\times 100$

**Table 5:** Management of different odontogenic cysts based on the provisional diagnosis with close follow up.

Details of Treatment Rendered		Radicular cyst N = 26	Dentigerous Cyst N = 9	Odontogenic kerato-cyst N = 17
Type of Surgical intervention	Marsupialization	1(3.85 %)	3(33.33 %)	0
	Enucleation	23 (88.4 %)	6(66.66 %)	12(70.58 %)
	Marsupialization + Enucleation	2(7.69 %)	0	5(29.41 %)
Under GA/LA	Local Anesthesia	25 (96.15 %)	8(88.88 %)	13(76.47 %)
	General Anesthesia	1 (3.85 %)	1(11.11 %)	4 (23.52 %)
Status succedaneous tooth	Erupted spontaneously	18 (69.23 %)	5(55.55 %)	0
	Required orthodontic traction	0	1(11.11 %)	6(35.29 %)
	Extracted during enucleation	6 (23.07 %)	3(33.33 %)	11(64.70 %)
Space maintainer Required		18 (69.23)	6(66.66 %)	6(35.29 %)
Follow-up	Mean ± SD in months	12.51 ± 5.64	15.22 ± 6.49	16.29 ± 6.93
Recurrence		0	0	4(23.52 %)

Radicular Cyst; Swelling associated with history of pain or extraction of decayed tooth, unilocular radiolucency at apex of concerned tooth with or without displacement of succedaneous tooth.

Dentigerous cyst; swelling with or without pain, well demarcated unilocular radiolucency located at the cemento-enamel junction of impacted tooth.

Odontogenic Keratocyst; swelling with or without pain, unilocular or multilocular radiolucency with well demarcated smooth or scalloped borders, may or may not been associated with the impacted tooth, and displacement of the suucedaneous tooth/teeth.

### 2.2 Screening for clinical and radiographic characteristics

After initial screening of the digital records, 84 cases were selected and among them, 23 cases were excluded due to incomplete digital record either in clinical, radiographic or histopathological, intervention or follow-up sections. Finally**,** 61 cases having complete digital record were selected for screening, for demographic details like age and sex (male, female), signs and symptoms, associated medical conditions, various clinical characteristics such as site of lesion anterior or posterior region of maxilla or mandible, whether the swelling is associated with primary teeth, status of primary teeth whether carious, pulpectomized as previous endodontic treatment can produce a periapical lesion due to insufficient treatment or deterioration, color and temperature of extra-oral swelling if present. The radiographic features include, radiographic investigations (Orthopantomogram, Cone-beamed computer tomography or any combination of them), borders of lesion (smooth, corticated, diffuse) locularity (unilocular, bilocular, or multilocular), radiographic size of lesion which was calculated from radiographic images and widest diameter in the axial view (<10 mm, 10–20 mm, 20–30 mm, >30 mm) was taken into consideration, status of associated succedaneous teeth (displaced, resorbed), and color of aspirate. The provisional diagnosis based on the above-mentioned findings made was also recorded. Note that no specify standardized OPG/CBCT protocols were used across cases, due to the existed variability.

### 2.3 Treatment and calculation of “discrepancy index”

The type of intervention, salvage of succedaneous teeth, definitive diagnosis after histopathological analysis, number of procedures, space maintainer given or not, eruption status of succedaneous teeth, procedure done under local anesthesia or general anesthesia, follow-up period (carried out by the concerned consultant), and recurrence (if the lesion recurs as the same site the characteristic features either clinical or radiographic) were also recorded based on the clinical and radiographic findings. For the calculation of the discrepancy between clinical and histopathological diagnosis (given by different pathologist who were blinded to the provisional diagnosis) of the radicular cyst, dentigerous cyst, and odontogenic kerato-cyst the “Discrepancy Index”^13^ was calculated by the given formula:

$$Discrepancy\;Index\left(DI\right)=\frac{Number\;of\;incompatible\;cases}{Total\;number\;of\;cases\;in\;the\;study}\times 100$$

### 2.4 Statistical analysis

Data was entered and analyzed using openEpi online software. For descriptive analysis, the ANOVA and Chi-square test were used to determine any significant differences between the two methods of assessment. Significance level was set at 5 % (p ≤ 0.05).

## 3 Result and observations

### 3.1 Demographic details (Table 1)

The demographic details retrieved from the digital records revealed that 61 cases of various cystic conditions were identified. Among them, the dentigerous cyst constituted 14.7 % (9cases), radicular cyst constituted 42.6 % (26cases), and Odontogenic kerato-cyst constitutes 27.86 % (17 cases) with the mean age (in years) of reporting 9.55 ± 3.16, 9.00 ± 2.79, and10.06 ± 2.43 respectively, without any significant difference among the three groups (p = 0.20, ANOVA test). The other cyst-like conditions constitute 14.75 % (9cases) which includes, AOT, CGCG, COF, FD, MNTI and ameloblastoma. Based on the gender, males (72.13 %) were found 2.59 times more affected as compared to females (27.86 %).

### 3.2 Clinical characteristics (Table 2):

**Fig. 2:**
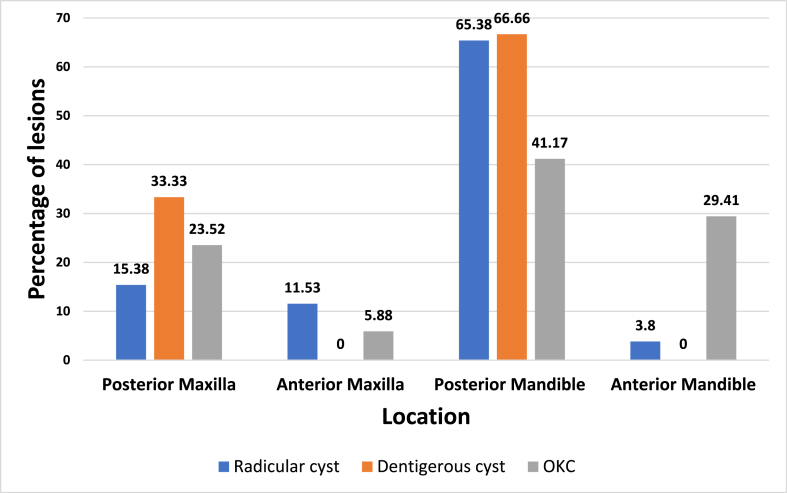
Percentage distribution of cystic lesions based on the anatomical site of jaws.

On screening of the digital records, It was observed that patients with radicular cyst mainly reported with the complaint of swelling (53.84 %) followed by a combination of pain and swelling (38.46 %), 7.6 % reported pain only. Similarly, in patients with dentigerous cysts, the main complaint with which the patient reported was observed to be swelling (66.66 %) followed by pain and swelling (33.33 %). Patients with Odontogenic keratocyst, it was observed that they reported with complaints of swelling (58.82 %) followed by pain and swelling (29.41 %) and followed by pain (11.775). Based on the location, it was observed that radicular cysts commonly occur in mandibular posterior region (65.38 %) followed by maxillary posterior region (15.38 %), maxillary anterior region (11.53 %) and least likely found in mandibular anterior region (3.8 %). Dentigerous cyst were commonly found in the mandibular posterior region (66.66 %) followed by maxillary posterior region (33.33 %). Odontogenic kerato-cysts were commonly found to be present in mandibular posterior region (41.17 %), followed by mandibular anterior region (29.41 %), maxillary posterior region (23.52 %) and least likely in maxillary anterior region (5.88 %) (Fig. 2). Looking the status of associated primary teeth, it was observed that in patients with radicular cysts, 19.23 % had history of primary tooth and 80.76 % had grossly decayed/pulpectomized teeth. In patients with dentigerous cysts, 44.44 % had a history of extraction of primary teeth, 55.55 % had decayed/pulpectomized teeth. In patients with Odontogenic kerato-cyst, 17.64 % had a history of extraction of primary tooth/teeth,35.29 % had decayed/pulpectomized primary tooth/teeth and 47.05 % had sound primary teeth.

**Fig. 3:**
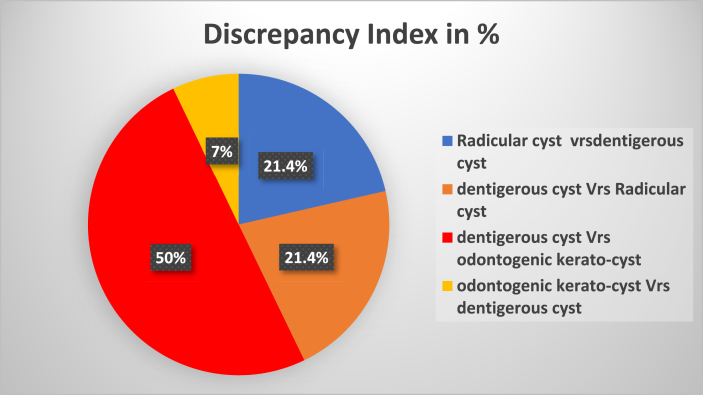
Represents the discrepancy in the diagnosis between clinical and histopathological assessment of odontogenic cystic lesions in mixed dentition period.

### 3.3 Radiographic characteristics (Table 3):

On screening of radiographic investigations, it was observed that various investigations that were carried out include OPG, CBCT, or combination of them. Radiographic evaluation revealed that the borders of radicular cysts were diffuse in 61.53 % and smooth in 38.46 %. In patients with dentigerous cysts, it was observed that the borders were smooth in 55.55 % and diffuse in 44.44 %. For the patients with Odontogenic keratocyst, the borders were corticated in 47.05 %, smooth in 41.17 % and diffuse I 11.76 %. (Due to the presence of smooth and borders in both dentigerous cyst, radicular cyst, and Odontogenic kerato-cyst, corticated borders in dentigerous cyst and Odontogenic keratocyst, the ambiguity could occur). Based on the locularity, it was observed that 96.15 % were unilocular and 3.84 % were multilocularity. In patients with dentigerous cysts, it was observed that 88.88 % were unilocular and 11.11 % were bilocular. 83.35 % Odontogenic kerato-cysts were observed to show unilocular presentation, 5.88 % were bilocular, and 11.76 % were multilocular. On observation of the status of succedaneous teeth, it was seen that in 7.69 % of cases of radicular cyst, succedaneous tooth/teeth was not present. In 11.53 % the tooth was involved but not displaced or resorbed, and in 80.76 % the succedaneous tooth was displaced from its anatomical location. In patients with dentigerous cysts, it was observed that in 88.88 % of cases the succedaneous tooth was displaced and in 11.11 % (1 case) dentigerous cyst was associated with the supernumerary tooth. In 88.23 % of Odontogenic kerato-cysts the involved succedaneous teeth/tooth were displaced and in 11.76 % cases succedaneous teeth/tooth were involved but not displaced. (Severity of displacement of the succedaneous tooth/teeth was not assessed). Based on the size of cystic lesion, 50 % of the radicular cyst were Class I (10–20 mm), 42.30 % were Class II (20–30 mm) and 7.69 % were class III (>30 mm). 22.22 % of dentigerous cyst were both class I, and class II and 55.55 % were class III. In Odontogenic keratocyst cases, 11.64 % were Class I, 52.94 % were Class II and 29.41 % were Class III. It was observed that there is no clear-cut demarcation between the radicular cyst, dentigerous cyst, and Odontogenic keratocyst based on the size of lesion, this could be other reason for the discrepancy of these lesions.

### 3.4 Discrepancy index: Table 4. Fig. 3

The discrepancy in diagnosis between the provisional diagnosis and definitive diagnosis of radicular cyst, dentigerous cyst, and Odontogenic keratocyst in the mixed dentition period was calculated by using “Discrepancy Index” as mentioned in methodology. Among 52 cases of radicular cyst, dentigerous cyst and Odontogenic keratocyst, 26.92 % (14 cases) show discrepancy. Among them the maximum discrepancy index was observed between dentigerous cysts and Odontogenic kerato-cysts i.e., 50 %, followed by radicular cyst and dentigerous cyst or vice-versa (21.42 %).

### 3.5 Management and follow-up: Table 5

Management of the cases was decided based on the diagnosis of the lesion with enucleation being the treatment of choice for the majority of the lesions. It was observed that marsupialization was carried out in 3.85 %of radicular cysts and 33.33 % of dentigerous cysts, enucleation was performed in 88.4 % of radicular cysts, 66.66 % of dentigerous cysts, and 70.58 % of Odontogenic keratocyst. In certain cases, the lesion that could not be enucleated due to extensive size or close relation to vital structures, especially in Odontogenic kerato-cyst (29.41 %) underwent two-step treatment of marsupialization followed by enucleation. The majority of radicular (96.15 %), dentigerous cysts (88.88 %), and Odontogenic keratocyst (76.47 %) were managed under local anesthesia and the rest were managed under general anesthesia due to larger extension and cooperation problems of patient (Treated under G. A, radicular cyst = 3.85 %; dentigerous cyst = 11.11 %; and Odontogenic keratocyst = 23.52 %). In 69.23 % of radicular cysts and 55.55 % of dentigerous cyst, the succedaneous teeth erupted spontaneously and 11.11 % (1 case) of dentigerous cyst and 35.29 % (6 cases) of OKC required orthodontic traction. During the surgical intervention, succedaneous teeth were extracted in 23.07 % of radicular cysts, 33.33 % of dentigerous cysts and 64.70 % of Odontogenic keratocyst. It was observed that in cases where succedaneous teeth were saved, space maintainer was given in the form of surgical stent made of self-curing acrylic that constitutes 69.23 % of radicular cyst, 66.66 % of dentigerous cyst and 35.29 % of Odontogenic keratocyst. The mean follow-up period of radicular cysts, dentigerous cysts and OKCs observed was 12.51 ± 5.64 months, 15.22 ± 6.49 and 16.29 ± 6.93 respectively. It was observed that only Odontogenic keratocyst show recurrence in 23.52 % (4Cases).

## 4 Discussion

### 4.1 Comparison with previous studies

Cystic lesions in the jaws share similarities in clinical and radiographic as well as symptomatology, especially between the dentigerous cyst and OKC.^15^ Radiographically, a dentigerous cyst usually presents as an unilocular and peri-coronal radiolucent lesion of an unerupted or impacted tooth in the jaws. The radiographic aspect of the OKC, when involving the crown of an enclosed tooth, can simulate a dentigerous cyst. Dentigerous cysts and OKC were the two most common types, accounting for over 95 % of developmental odontogenic cysts in pediatric patients.^16^, ^17^, ^18^, ^19^, ^20^ Therefore, knowledge of the incidence of dentigerous cysts and OKC and their more common location of presentation and age distribution will help practitioners to determine a more likely correct clinical early diagnosis**.** N. Lie et al., 2014 found that dentigerous cysts and OKC were the two most frequent developmental odontogenic cysts in children and adolescents, which constituted 97.8 %,^21^ in agreement with over 95 % reported in the literature mentioned above.^16^, ^17^, ^18^, ^19^ In the present study, the pattern of occurrence of odontogenic cysts observed was radicular cyst > odontogenic cyst > dentigerous cyst among the pediatric age group. The possible reasons for highest percentage of occurrence of radicular cyst could be; dental neglect in pediatric age group, myths regarding the primary teeth like they will shed after and new teeth will come; deficient number of pediatric dental specialist. Many studies involving patients of all ages have demonstrated that radicular cysts were the most common odontogenic cyst, followed by dentigerous cysts.^22^^,^^23^ However, it has been reported that dentigerous cysts are most common odontogenic cyst seen in permanent dentition.^24^^,^^25^ Based on the gender overall, males were found 2.59 times more affected as compared to females similar to the findings of N. Lie et al., 2014 who had reported males more affected than females and the reason being unknown.^21^ According to Benn and Altini et al. (2014),^24^ they are most closely related to mandibular third molars, followed by maxillary canines and mandibular second premolars. Sohan C. et al. (2022)^25^ reported that dentigerous cysts were more common in the anterior maxilla region (59.4 %) because mandibular third molars were not observed in most children*.* In the absence of mandibular third molars, 49.3 % of the dentigerous cysts were associated with impacted maxillary canines, followed by 21.7 % with impacted mandibular second premolars.^25^ Contrary to the observations of Sohan C. et al. (2022),^25^ in the present study, 65.38 % of radicular cysts were observed in the mandibular posterior region. With regard to anatomic location of the jaws, little information exists in a pediatric population. In our study, it was observed that all odontogenic cysts were predominantly located on the mandible. N. lie et al. (2014) On the other hand, cysts in the mandibular posterior region have a high probability of being radicular cysts.^21^ Considering that radicular cysts are associated with dental caries and inflammation, cystic lesions in the presence of dental caries or endodontic treatment as observed in the present study are likely to be diagnosed as radicular cysts. Although periapical lesions in radicular cysts are due to inflammatory conditions, they do not always cause pain.^26^ Most patients were asymptomatic for both dentigerous (98.6 %) and radicular cysts (61.1 %). Dentigerous cysts are generally asymptomatic. However, when infections affect dentigerous cysts, the size can be increased by expansion of the epithelial lining the cortical bone, resulting in pain and complications, such as pathologic fracture.^27^^,^^28^ The mean diameters of dentigerous and radicular cysts were 14.80 mm and 16.38 mm, respectively, dentigerous cysts have been found to be greater than 5 mm.^29^ In other studies, a diameter size of 10 mm was used as the landmark in the analysis of the size of odontogenic cysts.^30^ In the present study, no significant difference in the size of lesion was observed between dentigerous and radicular cysts. Sumer M et al. (2007)^31^ also reported that dentigerous cysts are often related to delayed eruption of the succeeding permanent tooth. The mechanism behind the failure of eruption of the succeeding permanent tooth in dentigerous cysts is unknown. In the present study, we did not assess the severity of the displaced succedaneous teeth. However, it was observed that in most of radicular cysts and dentigerous cysts, the succedaneous teeth erupt spontaneously. The succedaneous teeth which were extracted were mostly associated with the OKCs and the reason being the aggressive intervention. The other reasons for extraction of succedaneous teeth include lack of space for the lack of space for eruption, severe displacement of succedaneous tooth, and for orthodontic treatment needs. In a study by Zaib N. et al. (2023)^13^ he reported that most cases that showed discrepancy belonged to the neoplastic/non-neoplastic category, especially oral squamous cell carcinoma, which came out chronic inflammation not otherwise specified (NOS), Keratosis, dysplasia etc. The category included lesions that were both benign/malignant on clinician suspicion and turned out to be non-neoplastic on histology. A diagnostic discrepancy was also observed in OKC for dentigerous/ameloblastoma or vice versa. In the present study, it was observed that a maximum discrepancy index was observed between dentigerous cysts and OKCs, i.e., 50 %. This could be attributed to similar clinical and radiographic features of these odontogenic lesions. The discrepancy index observed between radicular cyst and dentigerous cyst or Vice-versa was (21.42 %), this could be due to the presence of follicles of succedaneous teeth present adjacent to the cystic lesion, association of dentigerous cyst below the carious teeth, and also may be due to large size of radicular cyst or small size of dentigerous cyst. Odontogenic cystic lesions may be treated by either complete surgical enucleation with or without peripheral ostectomy or by marsuplisation or marsuplisation followed by enucleation in order to enhance a gradual diminishing process utilizing the tendency of peripheral bone to centripetally grow into the cavity center following relief of the internal hydrostatic pressure caused by the cystic content. Marsupialization is a well-accepted technique for the primary treatment of odontogenic cysts. Previous studies of decompression and marsuplisation as a treatment for odontogenic cysts included all age groups.^32^^,^^33^ In a study by Dror M. Allon et al. (2014),^34^ out of the 32 lesions studied, 53 % were treated by decompression alone without evidence of recurrence after at least one year of follow-up. Nakamura et al. (2002) treated 28 odontogenic lesions by marsupliazation alone and found complete disappearance of the cysts in 17 % of the cases and decompression time for OKC/KOT averaged 23.5 months in a mostly adult group.^35^ Pogrel et al. (2004) allowed 7–19 months for marsupialization of OKC in a 10 patients' study group.^33^ In the present study, it was observed that most of the odontogenic cystic lesions were enucleated. In certain cases, the lesion that could not be enucleated due to extensive size or close relation to vital structures, especially in Odontogenic kerato-cyst underwent two-step treatment of marsupialization followed by enucleation. Only marsupialization was carried out in a few cases of radicular cysts and dentigerous cysts. Only OKC shows recurrence in 23.52 % of lesions in the known cases of Gorlin- Golts syndrome. This could be due to the inherent capability of OKC lesion for recurrence due to the presence of daughter cysts associated with the main lesion and due to the occurrence of multiple OKC in the above-mentioned syndrome and others may be due to salvage of succedaneous tooth that could have harbored daughter cyst. Findings from various interventions suggest no statistically significant association between recurrence rate and treatment method applied to related teeth.^36^ In the present study, during the surgical intervention, succedaneous teeth were extracted in 64.70 % of OKCs. The majority of radicular, dentigerous cysts, and OKCs were managed under local anesthesia and the rest were managed under general anesthesia due to larger extension and cooperation problems of patient.

### 4.2 Clinical implications

The clinical consequances of higher discrepancy in diagnosis between dentigerous cyst and OKCs could be; overtreatment of dentigerous cyst like peripheral ostectomy, use of Carnoys solution, extraction of adjacent tooth that is in proximity of cyst as observed in ᷉ 33 % of DCs, and under treatment of OKCs like salvage of adjacent tooth(35 %) that needs more aggressive intervention in order to prevent recurrence. Similarly, a higher discrepancy in diagnosis between radicular cyst and dentigerous cyst could lead to; under treatment of dentigerous cyst which in turn could transform into ameloblast, overtreatment of radicular cyst that can lead to the iatrogenic defect in the developing succedaneous tooth bud.

### 4.3 Novelty of the study

The long duration of this study significantly enhances the robustness and relevance of the data collected, offering comprehensive longitudinal insights into diagnostic discrepancies in pediatric dentistry. By specifically focusing on diagnostic discrepancies within mixed dentition, this research makes a unique contribution to the field. The use of both Orthopantomography (OPG) and Cone Beam Computed Tomography (CBCT) further enriches the diagnostic evaluation, allowing for a more thorough assessment and better comparability of findings. Additionally, the quantification of diagnostic mismatches through a formal index represents a methodological strength that is rarely reported in the literature, adding rigor and precision to the analysis. Importantly, the practical implications for treatment planning are significant; the findings can aid clinicians in making informed decisions and avoiding unnecessary interventions in children, ultimately leading to improved patient care and outcomes.

### 4.4 Limitations

The present study has several limitations that must be acknowledged. Firstly, the retrospective nature of the study design may introduce selection bias and result in incomplete information, which can affect the reliability of the findings. Additionally, the small sample size within each category of cystic lesions limits the generalizability of the results, making it difficult to apply the conclusions to a broader population. Furthermore, there is heterogeneity in the radiographic investigations utilized, which could lead to significant discrepancies in the data. This variability may stem from the use of both 2D and 3D imaging techniques, coupled with the absence of a standardized protocol for radiographic imaging. Another critical limitation is the histopathological diagnosis being conducted by different investigators, which introduces inter-observer variability and may impact the consistency of the results. Lastly, the average follow-up period of approximately 12–16 months may not be sufficient to capture all potential recurrences, particularly for odontogenic keratocysts (OKCs), which could lead to an underestimation of recurrence rates.

## 5 Conclusions

In conclusion, accurately diagnosing dentigerous cysts, radicular cysts, and odontogenic keratocysts (OKCs) in mixed dentition is challenging due to overlapping clinical and radiographic features, particularly in larger lesions. Histopathological examination is essential for definitive treatment, as misdiagnosis can lead to conservative approaches that may result in recurrence of OKCs or unnecessary extractions of dentigerous cysts. Notably, our study suggests that infections from carious teeth may trigger dentigerous cyst formation, highlighting the importance of caries prevention in primary teeth. However, the study's retrospective design and limited sample size restrict its generalizability. Future multicenter prospective studies are needed to establish standardized diagnostic protocols, which could enhance diagnostic accuracy and improve patient outcomes.

## Ethical clearance

Editing and removal of confidential content was done and ethically cleared by the institute.

## Consent form

Present study does not require patient consent as the patient details were kept confidential.

## Source of funding

No source of Funding

## Declaration of competing interest

Disparity in diagnosis and characteristics of odontogenic cystic lesions in mixed dentition period; A retrospective study.

There are no conflicts of interest.

## References

[1] Guler N., Comunoglu N., Cabbar F. (2012). Ki-67 and mcm-2 in dental follicle and odontogenic cysts: the effects of inflammation on proliferative markers. Sci World J.

[2] Mass E., Kaplan I., Hirshberg A. (1995). A clinical and histopathological study of radicular cysts associated with primary molars. J Oral Pathol Med.

[3] Bernardi L., Visioli F., Nör C. (2015). Radicular cyst: an update of the biological factors related to lining epithelium. J Endod.

[4] Noujeim Z., Nasr L. (2021). The prevalence, distribution, and radiological evaluation of dentigerous cysts in a Lebanese sample. Imaging Sci Dent.

[5] Johnson N.R., Gannon O.M., Savage N.W., Batstone M.D. (2014). Frequency of odontogenic cysts and tumors: a systematic review. J Investig Clin Dent.

[6] Shear M. (1994). Developmental odontogenic cysts. An update. J Oral Pathol Med.

[7] Daley T.D., Wysocki G.P. (1995). The small dentigerous cyst. A diagnostic dilemma. Oral Surg Oral Med Oral Pathol Oral Radiol Endod.

[8] Narang R.S., Manchanda A.S., Arora P., Randhawa K. (2012). Dentigerous cyst of inflammatory origin-a diagnostic dilemma. Ann Diagn Pathol.

[9] Speight P.M., Takata T. (2018). New tumour entities in the 4th edition of the World Health Organization Classification of Head and Neck tumours: odontogenic and maxillofacial bone tumours. Virchows Arch.

[10] Dammer R., Niederdellmann H., Dammer P., Nuebler-Moritz M. (1997). Conservative or radical treatment of keratocysts: a retrospective review. Br J Oral Maxillofac Surg.

[11] Ahlfors E., Larsson A., Sjögren S. (1984). The odontogenic keratocyst: a benign cystic tumor?. J Oral Maxillofac Surg.

[12] Stoelinga P.J. (2001). Long-term follow-up on keratocysts treated according to a defined protocol. Int J Oral Maxillofac Surg.

[13] Zaib N., Maqsood A., Ghayas S., Ansari F., Kayani A., Masood R. (2023). Analysis of discrepancy index between clinical and histopathological diagnosis of oral lesions. Asian Pac J Cancer Prev APJCP.

[14] Vandenbroucke Jan P., von Elm Erik, Altman Douglas G. (2014). Strengthening the reporting of observational studies in Epidemiology (STROBE): explanation and elaboration. Int J Surg.

[15] de Avila E.D., de Molon R.S., Massucato E.M., Hochuli-Vieira E. (2009). Relationship between the prevalence of the dentigerous cyst and the odontogenic keratocyst tumor and the current etiologic hypothesis. J Craniofac Surg.

[16] Jones A.V., Craig G.T., Franklin C.D. (2006). Range and demographics of odontogenic cysts diagnosed in a UK population over a30-year period. J Oral Pathol Med.

[17] Ochsenius G., Escobar E., Godoy L., Peñafiel C. (2007). Odontogenic cysts: analysis of 2,944 cases in Chile. Med Oral Patol Oral Cir Bucal.

[18] de Souza L.B., Gordón-Núñez M.A., Nonaka C.F., deMedeiros M.C., Torres T.F., Emiliano G.B. (2010). Odontogenic cysts: demographic profile in a Brazilian population over a 38-year period. Med Oral Patol Oral Cir Bucal.

[19] Tekkesin M.S., Olgac V., Aksakalli N., Alatli C. (2012). Odontogenic and nonodontogenic cysts in Istanbul: analysis of 5088 cases. Head Neck.

[20] Pechalova P.F., Bakardjiev A.G., Beltcheva A.B. (2011). Jaw cysts at children and adolescence: a single-center retrospective study of 152 cases in southern Bulgaria. Med Oral Patol Oral Cir Bucal.

[21] Li N., Gao X., Xu Z. (2014). Prevalence of developmental odontogenic cysts in children and adolescents with emphasis on dentigerous cyst and odontogenic keratocyst (keratocystic odontogenic tumor). Acta Odontol Scand.

[22] Kammer P.V., Mello F.W., Rivero E.R.C. (2020). Comparative analysis between developmental and inflammatory odontogenic cysts: retrospective study and literature review. Oral Maxillofac Surg.

[23] Soluk Tekkesin M., Tuna E.B., Olgac V., Aksakallı N., Alatlı C. (2016). Odontogenic lesions in a pediatric population: review of the litera ture and presentation of 745 cases. Int J Pediatr Otorhinolaryngol.

[24] Benn A., Altini M. (1996). Dentigerous cysts of inflammatory origin. A clinicopathologic study. Oral Surg Oral Med Oral Pathol Oral Radiol Endod.

[25] Sohn C., Ryu J., Nam I., Shin S.H., Lee J.Y. (2022). Cystic lesion between a deciduous tooth and the succeeding permanent tooth: a retrospective analysis of 87 cases. J Korean Assoc Oral Maxillofac Surg.

[26] Boudaoud Z., Maou S., Badi Y. (2016). Radicular cyst on deciduous mo lar or dentigerous cyst on permanent tooth?. Int J Dent Oral Sci.

[27] Mohan K.R., Natarajan B., Mani S., Sahuthullah Y.A., Kannan A.V., Doraiswamy H. (2013). An infected dentigerous cyst associated with an impacted permanent maxillary canine, inverted mesiodens and impacted supernumerary teeth. J Pharm BioAllied Sci.

[28] Patil A.S., Jathar P.N., Panse A.M., Bahutule S.R., Patil R.U., Patil M. (2019). Infected dentigerous cyst and its conservative management: a re port of two cases. Int J Clin Pediatr Dent.

[29] Batra P., Roychoudhury A., Balakrishan P., Parkash H. (2004). Bilateral den tigerous cyst associated with polymorphism in chromosome 1qh+. J Clin Pediatr Dent.

[30] Bilodeau E.A., Collins B.M. (2017). Odontogenic cysts and neoplasms. Surg Pathol Clin.

[31] Sumer M., Baş B., Yildiz L. (2007). Inferior alveolar nerve paresthesia caused by a dentigerous cyst associated with three teeth. Med Oral Patol Oral Cir Bucal.

[32] Anavi Y., Gal G., Miron H., Calderon S., Allon D.M. (2011). Decompression of odontogenic cystic lesions: clinical long-term study of 73 cases. Oral Surg Oral Med Oral Pathol Oral Radiol Endod.

[33] Pogrel M.A., Jordan R.C. (2004). 'Marsupialization as a definitive treatment for the odontogenic keratocyst. J Oral Maxillofac Surg.

[34] Allon D.M., Allon I., Anavi Y. (2014). Decompression as a treatment of odontogenic cystic lesions in children. J Oral Maxillofac Surg.

[35] Nakamura N., Mitsuyasu T., Mitsuyasu Y., Taketomi T., Higuchi Y., Ohishi M. (2002). Marsupialization for odontogenic keratocysts: long-term follow-up analysis of the effects and changes in growth characteristics. Oral Surg Oral Med Oral Pathol Oral Radiol Endod.

[36] Karaca Ç., Dere K.A., Er N. (2018). Recurrence rate of odontogenic keratocyst treated by enucleation and peripheral ostectomy: retrospective case series with up to 12 years of follow-up. Med Oral Patol Oral Cir Bucal.

